# Variety more than quantity of fruit and vegetable intake varies by socioeconomic status and financial hardship. Findings from older adults in the EPIC cohort^☆^

**DOI:** 10.1016/j.appet.2014.08.038

**Published:** 2014-12-01

**Authors:** Annalijn I. Conklin, Nita G. Forouhi, Marc Suhrcke, Paul Surtees, Nicholas J. Wareham, Pablo Monsivais

**Affiliations:** a MRC Epidemiology Unit, University of Cambridge School of Clinical Medicine, Cambridge Biomedical Campus, UK; b Centre for Diet and Activity Research University of Cambridge School of Clinical Medicine, Box 285 Institute of Metabolic Science, Cambridge, CB2 0QQ, United Kingdom; c Faculty of Medicine and Health Sciences, University of East Anglia, Norwich, UK; d Department of Public Health and Primary Care, Institute of Public Health, University of Cambridge, UK

**Keywords:** Healthy eating, Variety, Socioeconomic inequality, Financial hardship, Aging, UK

## Abstract

*Background:* Beyond quantity, variety of fruit and vegetable (FV) intake prevents chronic conditions and is widely recommended as critical to healthful eating. FV consumption is socially patterned, especially for women, but little is known about multiple economic determinants of variety or whether they differ from those of quantity. *Objective:* To examine socioeconomic status and financial hardships in relation to variety and quantity of FV intakes among older British women and men. *Methods:* Cross-sectional study of 9580 adults (50–79 years) in the nationally representative EPIC cohort who responded to a postal Health and Life Experiences Questionnaire (1996–2000) and Food Frequency Questionnaire (1998–2002). Variety counted unique items consumed (items/month) and quantity measured total intake (g/day). *Results:* No consistent differences by any economic factor were observed for quantity of fruits or vegetables, except education in men. Lower education, lower social class and renting were independently associated with lower fruit variety and vegetable variety (*p*-trend < 0.001), with differences stronger in men. Mean vegetable variety differed between top and bottom social classes by 2.9 items/month for men and 2.5 for women. Greater financial hardships were also independently associated with lower variety, with differences stronger in women for fruits and in men for vegetables. *Conclusions:* British older adults reporting greater economic disadvantage consistently consumed fewer different fruits or vegetables, but not lower amounts. Further nutrition studies of the protective effects, and underlying mechanisms, of FV variety are warranted for addressing social inequalities in older adults' diet quality. Dietary guidance should separately emphasise variety, and interventions should aim to address financial barriers to older adults' consumption of diverse FV.

## Highlights

• Eating a variety of many fruits and vegetables is critical for healthy aging.
• Variety is as important as quantity of fruits and vegetables for disease prevention.
• Fruit and vegetable consumption is strongly graded by socioeconomic status.
• Variety was strongly patterned by socioeconomic status and by financial hardships.
• Economic inequalities were greater in women for fruit and men for vegetable variety.

## Introduction

Inadequate intakes of fruits and vegetables (FV) contribute to approximately 5% of excess deaths worldwide and higher risk of chronic conditions (World Health Organization, 2004). Beyond quantity, variety of FV is also important for supporting health and is widely recommended as critical to healthful eating (USDA, DHHS, 2010, World Health Organization, 2009). Several prospective studies have shown that higher FV variety reduced the risk of T2D and some cancers, independent of quantity of intake (Cooper et al, 2012, Jeurnink et al, 2012). A mixed diet overall might also help to reduce hospitalisations and utilisation of acute medical care among older adults (Lo, Wahlqvist, Chang, Kao, & Lee, 2013).

Greater food variety is desirable for preventing, or managing, chronic conditions because variety improves nutritional adequacy and diet quality by increasing a person's exposure to a wide range of nutrients and phytochemicals necessary to support normal physical functioning (Drescher et al, 2007, Foote et al, 2004). Thus, as the population ages, there is increasing importance for health policy and population-level strategies to promote consumption of more and varied FV so as to prevent the large and growing global burden of chronic conditions that impose substantial personal and social costs (World Health Organization, 2004, World Health Organization, 2009). Efforts to promote healthful eating, however, must be informed by evidence on the determinants of both quantity and variety of FV consumption since these measures have independent implications for health.

It is known that consumption levels of FV are strongly associated with socioeconomic status (SES) as a conventional proxy for economic resources measured by income, occupational grade, education, or wealth (Darmon & Drewnowski, 2008). Diet variety is also associated with socioeconomic factors (Ahn, Engelhardt, & Joung, 2006). Beyond standard SES measures, a person's financial situation, particularly in older ages, is also worth considering as a unique economic determinant of healthful eating since everyday financial troubles are not sufficiently characterised by conventional SES indicators (Sullivan, Turner, & Danziger, 2008). Moreover, self-reported economic hardships likely represent concrete financial strain that may exert a more direct influence on decisions about purchasing and consuming FV (Bihan et al., 2010). One study of overall financial hardship reported associations with healthy food habits in working Finnish adults, independent of education, occupational grade, income and home-ownership (Lallukka, Laaksonen, Rahkonen, Roos, & Lahelma, 2006). Yet, a holistic assessment of diverse economic factors influencing diet among older populations remains neglected in the literature (Payette & Shatenstein, 2005). The absence of studies on healthful eating examining different types of financial hardships is problematic because older people can experience some hardships more than others (Kahn, Pearlin, 2006, Office of National Statistics, 2011).

To fill this gap, we investigated three conventional SES indicators and three financial hardship measures in relation to both variety and quantity of fruits and/or vegetables in older British women and men.

## Subjects and methods

We used data collected in the population-based European Prospective Investigation in Cancer cohort study in Norfolk, UK (EPIC-Norfolk) (Bingham et al., 2001). EPIC-Norfolk recruited 25,639 men and women aged 39–79 from age–sex registers of general practices (99.7% white), who attended a first health check at entry (1993–1997). Because this study is focused on economic circumstances of adults near the end of working life and beyond, the eligible sample was 20,274 over-50s who were similar to the total cohort at entry regarding socio-demographics and health behaviours. All volunteers gave written informed consent and the study was approved by the Norwich district ethics committee.

Three conventional indicators of SES, self-reported at cohort entry, were analysed. Educational attainment was in four groups, based on years in education: no qualification (<11 years), O-level (11 years), A-level (≥13 years but no degree), degree (≥16 years). Social class was based on six hierarchical categories of the Registrar General's classification scheme of occupations (professional, managerial and technical, skilled non-manual, skilled manual, partly skilled, and unskilled) (Office of Population Censuses and Surveys, 1992). Self-reported accommodation (home-owner, public renting and private renting) was also examined as a conventional SES proxy since previous research documents the utility of home-ownership as a measure of wealth in older populations (Pollack et al., 2007), and wealth is known to influence older adults' diet (Maynard et al., 2006).

Financial hardship (FH) was assessed using a postal “Health and Life Experiences Questionnaire” (1996–2000) designed to assess social and psychological circumstances (Surtees & Wainwright, 2007). As previously examined in this sample (Conklin et al., 2013) and consistent with Pearlin's list of chronic strains on household economics (Pearlin & Schooler, 1978), three questions covered sufficiency of money to meet needs (more than enough, just enough, less than enough), frequency of not having enough money to afford adequate food or clothing (five responses, between ‘never’ and ‘always’), and difficulty paying bills (six responses, between ‘none’ and ‘very great’). Responses ‘often’ and ‘always’, or ‘great’ and ‘very great’, were combined for analysis to increase numbers. Completed responses from over-50s ranged between 17,953 and 17,998 depending on the individual question.

A Food Frequency Questionnaire – previously validated by comparing to a 16-day weighed food record (Bingham et al., 1994) and nutrient biomarkers (Bingham et al., 2001) – was completed at the second health check (1998–2002). Participants (n = 9933) reported their consumption of a pre-specified number of fruits (n = 11) (whole item or medium serving), and vegetables (n = 26) (medium serving) over the last year, with nine standard response categories (between never or less than once/month and ≥6/day) (Willett, 2013). FFQ respondents with extreme estimated energy intakes (defined as top and bottom 0.5 percentile of energy intake relative to basal metabolic rate values) were excluded (n = 353). Average daily consumption of each fruit and vegetable item (g/day) was estimated from self-reported frequencies and imputed standard portion sizes using an established method (Welch, Luben, Khaw, & Bingham, 2005). A continuous variable for quantity was derived by summing the total amount of fruits, vegetables and both. Variety of fruit and/or vegetable intake was a sum of the total number of unique items consumed, irrespective of quantity (>0 g/day), which corresponded to response categories of at least 1–3/month. This scoring method followed similar approaches previously demonstrated for reduced risk of chronic diseases in this cohort (Cooper et al, 2012, Jeurnink et al, 2012), and reflected the minimum 2 weeks needed for a person to exhaust the variety of their food repertoire (Drewnowski, Henderson, Driscoll, & Rolls, 1997). Continuous scores were derived for variety (items/month) of fruit (range 0–11), vegetable (0–26), and combined (0–37). Other studies have demonstrated the reproducibility and validity of variety scores for nutritional adequacy in older populations (Bernstein et al, 2002, Drewnowski et al, 1997).

Concurrent socio-demographic variables included: self-rated general health status (excellent, good, moderate, poor), smoking status (current, former, never), marital status (married/living as married, single, widowed, separate, divorced), and total energy intake (kcal/day). Height and weight measured at entry were used to calculate BMI (kg/m^2^) and baseline physical activity level was self-reported. Age and sex were also included as covariates. The analysed sample had almost complete information and included over-50s who responded to SES and FH questions, had key covariates and plausible dietary data at follow-up, averaging 18 months (range: 8413–8425) (Appendix S1A); characteristics and lifestyle were similar to cohort participants.

Descriptive statistics summarised socio-demographic characteristics and crude mean FV quantity and variety across levels of multiple economic conditions. Multivariable linear regression models assessed cross-sectional associations between each economic variable and both dietary outcomes, adjusting for total energy intake (kcal/day), age, and marital status – known confounders associated with economic disadvantage and diet (Conklin et al, 2013, Irz et al, 2013). Regression coefficients were then used for post-estimation calculation of adjusted means and 95% confidence intervals (CI95) (model A) for each level of SES or FH as recommended in the literature (Braveman et al., 2005). In addition, each SES indicator was further adjusted for all FH measures, and vice versa (model B), and thus the remaining mean variety or quantity of fruits and/or vegetables was interpreted as independent effects of a given economic exposure. Main analyses were a priori performed separately for women and men to examine gender-specific associations and therefore models were specified with an interaction term between the sex and exposure variables.

Sensitivity analyses of both covariate- and SES-adjusted models included additional conditioning on quantity (for variety as the independent variable), variety (for quantity as the independent variable), or other concurrent lifestyle factors (total alcohol (continuous); physical activity and energy expenditure (continuous); and smoking status (current, ex- or never)). We also considered potential confounding by poor/moderate health status and thus re-examined independent associations after excluding over-50s who rated their health as poor or moderate at cohort entry. In post-hoc analyses, we evaluated the statistical significance of possible linear trend in the results for social class, education, and FH by using linear contrast in coefficients from stratified covariate-adjusted models and formulae were specific to each variable's number of levels (e.g. 5 for difficulty paying bills). We used the statistic to evaluate differences in group-specific means for the accommodation variable. Statistical analyses were conducted using Stata version 12.1.

## Results

Our sample of 55% women averaged 62 years, with 83% reporting good/excellent general health and 51% having ever smoked. For the whole sample 13% were educated to degree-level, although 15% of men and 11% of women had degree education. The top-two social classes comprised 46% of the sample; more women (4%) than men (2%) had unskilled occupations. Men and women were generally overweight at follow-up (26.8 kg/m^2^ (SD 3.3) and 26.7 kg/m^2^ (SD 4.4), respectively). Variety scores were normally distributed, with few over-50s reported no consumption (0 g/day) of any fruit (n = 55) or vegetable (n = 6) item and therefore scored zero for variety.

Some socio-demographic characteristics and self-rated general health of the sample varied across levels of SES and FH (Table 1). Crude mean scores for variety of combined FV decreased across increasing levels of hardship. FV quantity was consistently lowest only for the greatest hardship category, but no clear pattern of differences in mean energy intake was seen across economic conditions.

**Table 1:** Characteristics of older adults in the EPIC-Norfolk study by levels of socioeconomic status or financial hardship.^a^

	Women (%)	Mean (SD) Age	Mean (SD) BMI (kg/m^2^)	Physical inactivity (%)	Not married (%)	Poor/ moderate health (%)	Ever smoker (%)	Mean (SD) Kcal/d	Mean (SD) FV variety (items/mo)	Mean (SD) FV quantity (g/d)
**Social class (n** **=** **8535)**										
Professional (n = 634)	50	62 (7)	25.8 (3.4)	60	13	10	45	1936 (528)	25 (5)	554 (251)
Managerial and technical (n = 3354)	53	62 (7)	26.0 (3.5)	60	18	14	50	1959 (541)	25 (5)	555 (254)
Skilled non-manual (n = 1509)	65	63 (7)	26.0 (3.6)	66	30	17	49	1919 (549)	23 (6)	540 (251)
Skilled manual (n = 1734)	53	61 (7)	26.5 (3.7)	52	14	19	56	1967 (564)	23 (6)	534 (258)
Partly skilled (n = 1064)	54	62 (7)	26.7 (4.0)	53	21	21	55	1970 (595)	22 (6)	532 (293)
Unskilled (240)	65	62 (7)	27.2 (4.5)	51	40	23	49	1959 (608)	21 (6)	519 (276)
**Education (n** **=** **8678)**										
Degree (n = 1183)	48	63 (7)	25.8 (3.5)	57	20	10	44	1939 (552)	25 (5)	552 (250)
A-level (n = 3600)	49	61 (7)	26.1 (3.6)	56	19	15	53	1967 (552)	24 (5)	544 (255)
O-level (n = 876)	61	62 (7)	26.0 (3.7)	64	17	15	49	1937 (524)	24 (6)	537 (242)
No qualification (n = 3019)	63	61 (7)	26.6 (3.8)	60	23	20	78	1949 (574)	22 (5)	542 (275)
**Home-ownership (n** **=** **8538)**										
Owner–occupier (=7878)	55	62 (7)	26.1 (3.6)	58	18	15	51	1958 (555)	24 (5)	545 (255)
Renter, private (n = 217)	56	64 (8)	26.4 (3.5)	54	39	18	54	1939 (561)	22 (6)	527 (276)
Renter, public (n = 443)	62	64 (7)	27.2 (4.3)	66	45	31	56	1909 (590)	21 (6)	524 (335)
**Having enough money for needs (n** **=** **8413)**										
More than enough (n = 1640)	53	61 (7)	25.7 (3.6)	59	16	10	46	1942 (532)	24 (5)	539 (243)
Just enough (n = 6011)	56	62 (7)	26.2 (3.6)	58	20	16	51	1959 (561)	24 (6)	547 (262)
Less than enough (n = 762)	53	61 (7)	27.2 (4.4)	60	31	28	62	1931 (564)	22 (6)	530 (282)
**Frequency of not having enough money for food or clothing (n** **=** **8417)**										
Never (n = 5197)	54	62 (7)	26.0 (3.5)	58	18	13	49	1954 (545)	24 (6)	545 (258)
Seldom (n = 1869)	56	62 (7)	26.4 (3.8)	58	21	18	52	1964 (558)	24 (6)	549 (253)
Sometimes (n = 1005)	59	61 (7)	26.7(4.0)	57	24	21	53	1975 (590)	23 (6)	546 (284)
Often/Always (n = 346)	61	61 (7)	26.7(4.3)	62	36	32	59	1823 (561)	21 (6)	502 (257)
**Difficulty paying bills (n** **=** **8425)**										
None (n = 5151)	55	63 (7)	26.0 (3.5)	59	17	13	50	1952 (549)	24 (6)	547 (257)
Very little (n = 1991)	54	62 (7)	26.3 (3.6)	56	21	17	52	1965 (559)	24 (5)	544 (257)
Slight (n = 602)	56	61 (7)	26.6 (3.9)	56	22	21	55	1939 (576)	24 (6)	544 (270)
Some (n = 571)	60	61 (7)	27.0 (4.5)	58	33	28	56	1940 (563)	23 (6)	524 (275)
Great/Very great (n = 110)	60	60 (7)	27.6 (4.5)	58	35	35	68	1969 (624)	21 (6)	531 (307)

Note: FV, fruit and vegetable.

a Measurement time-points were: sex, age, education, occupational social class, BMI, physical activity (1993–1997); accommodation and financial hardship measures (1996–2000); self-rated general health, smoking status, marital status and dietary intake (1998–2002).

### Fruit and vegetable intake by conventional SES groupings

We found a consistent difference by SES in the adjusted mean scores for fruit variety, but less consistently for fruit quantity (Table 2, model A). Fruit quantity varied significantly across levels of education and social class only. Clear and strong differences by SES were also observed in vegetable variety, but not in quantity, for both women and men (Table 3, model A). The only exception was educational differences in vegetable quantity for men. At any level of conventional SES, women consumed greater variety and quantity of fruits or vegetables than men, adjusted for total energy, age and concurrent marital status.

**Table 2:** Adjusted mean quantity and variety of fruit intakes by socioeconomic status among older adults in the EPIC-Norfolk study.

	Fruit quantity (g/d)				Fruit variety (items/mo)			
	Women		Men		Women		Men	
	Model A	Model B: + FH	Model A	Model B: + FH	Model A	Model B: + FH	Model A	Model B: + FH
**Social class**								
Professional	320 (300, 339)	321 (301, 342)	264 (244, 283)	263 (243, 283)	8.0 (7.8, 8.3)	8.0 (7.7, 8.2)	7.4 (7.2, 7.7)	7.4 (7.1, 7.6)
Managerial and technical	306 (235, 253)	306 (297, 314)	244 (235, 253)	242 (233, 252)	8.0 (7.9, 8.1)	8.0 (7.9, 8.1)	7.0 (6.9, 7.2)	7.0 (6.9, 7.1)
Skilled non-manual	295 (284, 306)	292 (281, 304)	236 (220, 251)	234 (218, 250)	7.6 (7.4, 7.7)	7.5 (7.4, 7.7)	6.6 (6.4, 6.8)	6.6 (6.4, 6.8)
Skilled manual	295 (284, 306)	294 (282, 306)	225 (214, 237)	227 (215, 240)	7.5 (7.3, 7.6)	7.5 (7.3, 7.7)	6.4 (6.2, 6.5)	6.4 (6.3, 6.6)
Partly skilled	295 (280, 309)	297 (282, 313)	228 (212, 243)	232 (215, 248)	7.5 (7.3, 7.6)	7.5 (7.3, 7.7)	6.2 (6.0, 6.4)	6.4 (6.2, 6.6)
Unskilled	287 (260, 313)	281 (252, 310)	209 (173, 246)	211 (172, 250)	7.0 (6.7, 7.4)	7.1 (6.7, 7.4)	6.1 (5.6, 6.5)	6.1 (5.6, 6.6)
*P-trend*	*0.021*	*0.019*	*0.001*	*0.006*	*<0.001*	*<0.001*	*<0.001*	*<0.001*
**Education**								
Degree	318 (303, 333)	317 (302, 333)	261 (247, 275)	258 (244, 273)	8.2 (8.1, 8.4)	8.2 (8.0, 8.4)	7.5 (7.3, 7.6)	7.4 (7.2, 7.6)
A-level	307 (299, 315)	306 (297, 314)	233 (225, 241)	233 (225, 242)	8.0 (7.9, 8.1)	7.9 (7.8, 8.1)	6.8 (6.7, 6.9)	6.8 (6.7, 6.9)
O-level	291 (277, 306)	291 (276, 307)	239 (220, 257)	234 (215, 254)	7.8 (7.6, 8.0)	7.8 (7.6, 7.9)	6.7 (6.5, 7.0)	6.7 (6.4, 6.9)
No qualification	292 (284, 299)	291 (282, 299)	231 (221, 241)	233 (223, 244)	7.3 (7.2, 7.4)	7.4 (7.2, 7.5)	6.3 (6.2, 6.4)	6.3 (6.2, 6.5)
*P-trend*	*0.001*	*0.002*	*0.001*	*0.01*	*<0.001*	*<0.001*	*<0.001*	*<0.001*
**Home-ownership**								
Owner–occupier	300 (294, 305)	299 (294, 305)	239 (233, 245)	238 (232, 245)	7.8 (7.7, 7.8)	7.8 (7.7, 7.8)	6.8 (6.7, 6.9)	6.8 (6.7, 6.9)
Renter, private	293 (260, 326)	296 (263, 328)	224 (187, 260)	225 (188, 262)	7.2 (6.8, 7.6)	7.3 (6.9, 7.7)	6.1 (5.6, 6.6)	6.2 (5.7, 6.6)
Renter, public	299 (277, 320)	302 (280, 324)	225 (197, 252)	229 (201, 257)	7.0 (6.7, 7.3)	7.1 (6.9, 7.4)	5.9 (5.6, 6.3)	6.0 (5.7, 6.4)
*P-difference*	*0.924*	*0.947*	*0.468*	*0.654*	*<0.001*	*<0.001*	*<0.001*	*<0.001*

Note: FH, financial hardship. Gender-specific means (CI95) obtained by multivariable linear regression analysis adjusted for total energy intake (kcal/d), baseline age (continuous), and concurrent marital status (categorical) (model A); then for FH (money for needs; frequency of insufficient money for food/clothing; difficulty paying bills) (model B). Model B numbers were: social class (8535); education (8678); home-ownership (8538).

**Table 3:** Adjusted mean quantity and variety of vegetable intakes by socioeconomic status among older adults in the EPIC-Norfolk study.

	Vegetable quantity				Vegetable variety			
	Women		Men		Women		Men	
	Model A	Model B: + FH	Model A	Model B: + FH	Model A	Model B: + FH	Model A	Model B: + FH
**Social class**								
Professional	278 (264, 291)	280 (266, 294)	261 (248, 274)	259 (246, 273)	17.6 (17.2, 18.0)	17.6 (17.2, 18.0)	17.2 (16.7, 17.6)	17.1 (16.6, 17.5)
Managerial and technical	291 (285, 297)	293 (287, 298)	266 (260, 272)	267 (261, 274)	17.2 (17.1, 17.4)	17.2 (17.1, 17.4)	16.6 (16.5, 16.8)	16.6 (16.4, 16.8)
Skilled non-manual	278 (270, 285)	279 (271, 286)	253 (243, 263)	254 (243, 265)	16.3 (16.1, 16.5)	16.3 (16.1, 16.5)	15.7 (15.3, 16.0)	15.7 (15.3, 16.0)
Skilled manual	287 (279, 294)	284 (275, 292)	255 (247, 263)	254 (246, 263)	15.9 (15.7, 16.2)	15.9 (15.7, 16.2)	15.0 (14.8, 15.3)	15.1 (14.8, 15.4)
Partly skilled	272 (262, 282)	271 (261, 281)	257 (247, 268)	256 (245, 267)	15.8 (15.5, 16.1)	15.8 (15.5, 16.1)	14.9 (14.5, 15.2)	15.0 (14.7, 15.3)
Unskilled	278 (260, 296)	267 (249, 288)	246 (221, 271)	244 (217, 271)	15.1 (14.5, 15.7)	15.2 (14.6, 15.8)	14.3 (13.5, 15.1)	14.4 (13.6, 15.2)
*P-trend*	*0.393*	*0.077*	*0.156*	*0.155*	*<0.001*	*<0.001*	*<0.001*	*<0.001*
**Education**								
Degree	282 (272, 292)	281 (271, 292)	263 (254, 273)	263 (253, 273)	17.7 (17.4, 18.0)	17.6 (17.3, 17.9)	17.1 (16.8, 17.4)	17.0 (16.7, 17.3)
A-level	288 (282, 293)	288 (282, 294)	264 (258, 269)	264 (258, 269)	17.1 (17.0, 17.3)	17.1 (17.0, 17.3)	16.1 (15.9, 16.3)	16.2 (16.0, 16.3)
O-level	277 (267, 287)	278 (267, 288)	254 (241, 266)	254 (240, 267)	16.7 (16.4, 17.0)	16.7 (16.4, 17.0)	16.3 (15.9, 16.7)	16.2 (15.8, 16.6)
No qualification	283 (277, 288)	281 (275, 287)	253 (246, 260)	254 (247, 261)	15.6 (15.5, 15.8)	15.7 (15.5, 15.8)	14.8 (14.6, 15.0)	14.9 (14.7, 15.2)
*P-trend*	*0.662*	*0.638*	*0.019*	*0.050*	*<0.001*	*<0.001*	*<0.001*	*<0.001*
**Home-ownership**								
Owner–occupier	285 (281, 288)	285 (281, 289)	261 (257, 265)	261 (257, 265)	16.7 (16.6, 16.8)	16.7 (16.6, 16.8)	16.0 (15.9, 16.2)	16.1 (15.9, 16.2)
Renter, private	283 (261, 306)	283 (260, 305)	247 (222, 272)	248 (222, 273)	16.0 (15.3, 16.7)	16.1 (15.4, 16.8)	15.2 (14.5, 16.0)	15.4 (14.6, 16.1)
Renter, public	267 (253, 282)	264 (249, 279)	251 (233, 270)	250 (231, 269)	14.8 (14.4, 15.3)	15.0 (14.5, 15.5)	13.9 (13.3, 14.5)	14.0 (13.4, 14.6)
*P-difference*	*0.111*	*0.050*	*0.308*	*0.276*	*<0.001*	*<0.001*	*<0.001*	*<0.001*

Note: FH, financial hardship. Gender-specific means (CI95) obtained by multivariable linear regression analysis adjusted for total energy intake (kcal/d), baseline age (continuous), and concurrent marital status (categorical) (model A); then for FH (money for needs; frequency of insufficient money for food/clothing; difficulty paying bills) (model B). Model B numbers were: social class (8535); education (8678); home-ownership (8538).

The relationships between each SES variable and each dietary outcome were similar in additional models further adjusting for financial hardships (FH) (model B). Only one gender-specific exception was noted: differences in adjusted means of vegetable quantity by type of accommodation became near-significant in women (*p*-trend = 0.05). Results for covariate- and FH-adjusted associations were repeated when fruit and vegetable intake was combined (Appendix S1B).

### Fruit and vegetable intake by financial hardship levels

In covariate-adjusted models, the relationship between FH and quantity of fruits also did not show clear inverse associations or differences by gender (Table 4, model A). By contrast, differences in adjusted mean fruit variety across levels of each measure of FH were seen in women and men. Results were similar for vegetable intake (Table 5, model A). Notably, women appeared to have stronger associations between FH and fruit variety, while men showed stronger hardship differences in relation to vegetable variety.

**Table 4:** Adjusted mean quantity and variety of fruit intakes by financial hardship among older adults in the EPIC-Norfolk study.

	Fruit quantity				Fruit variety			
	Women		Men		Women		Men	
	Model A	Model B: + SES	Model A	Model B: + SES	Model A	Model B: + SES	Model A	Model B: + SES
**Enough money for needs**								
More than enough	299 (287, 311)	295 (283, 308)	241 (228, 254)	237 (224, 250)	8.0 (7.8, 8.1)	7.8 (7.6, 7.9)	7.1 (6.9, 7.3)	6.9 (6.8, 7.1)
Just enough	300 (294, 306)	301 (295, 307)	236 (229, 243)	239 (232, 246)	7.7 (7.6, 7.8)	7.7 (7.7, 7.8)	6.7 (6.6, 6.8)	6.7 (6.7, 6.8)
Less than enough	297 (280, 315)	297 (279, 315)	237 (219, 256)	238 (219, 257)	7.2 (7.0, 7.4)	7.4 (7.1, 7.6)	6.3 (6.1, 6.6)	6.5 (6.3, 6.8)
*P-trend*	*0.754*	*0.961*	*0.766*	*0.912*	*<0.001*	*0.001*	*<0.001*	*0.023*
**Frequency of insufficient money for food/clothing**								
Never	301 (295, 308)	302 (295, 308)	241 (234, 248)	241 (234, 248)	7.8 (7.7, 7.9)	7.7 (7.7, 7.8)	6.8 (6.7, 6.9)	6.8 (6.7, 6.8)
Seldom	299 (288, 309)	300 (289, 311)	233 (220, 245)	236 (224, 249)	7.8 (7.7, 7.9)	7.9 (7.7, 8.0)	6.8 (6.6, 6.9)	6.9 (6.7, 7.0)
Sometimes	300 (286, 315)	300 (285, 315)	226 (209, 243)	229 (211, 247)	7.5 (7.3, 7.7)	7.6 (7.4, 7.8)	6.6 (6.4, 6.8)	6.8 (6.5, 7.0)
Often/Always	269 (245, 293)	268 (242, 293)	233 (203, 262)	230 (199, 261)	6.9 (6.6, 7.2)	7.1 (6.8, 7.4)	6.1 (5.7, 6.5)	6.3 (5.9, 6.7)
*P-trend*	*0.019*	*0.017*	*0.472*	*0.397*	*<0.001*	*<0.001*	*<0.001*	*0.039*
**Difficulty paying bills**								
None	303 (297, 310)	303 (296, 310)	242 (234, 249)	242 (235, 250)	7.8 (7.7, 7.8)	7.7 (7.6, 7.8)	6.8 (6.7, 6.9)	6.7 (6.7, 6.8)
Very little	294 (283, 305)	294 (283, 305)	233 (221, 244)	235 (223, 247)	7.8 (7.7, 8.0)	7.9 (7.7, 8.0)	6.8 (6.6, 6.9)	6.8 (6.7, 7.0)
Slight	300 (281, 320)	302 (282, 322)	227 (205, 249)	227 (205, 249)	7.6 (7.3, 7.8)	7.7 (7.4, 7.9)	6.9 (6.6, 7.2)	6.9 (6.6, 7.2)
Some	290 (271, 309)	292 (273, 312)	218 (194, 241)	221 (197, 245)	7.4 (7.1, 7.6)	7.5 (7.3, 7.8)	6.4 (6.1, 6.7)	6.5 (6.2, 6.8)
Great/Very great	261 (218, 304)	253 (209, 298)	248 (196, 301)	244 (190, 298)	6.6 (6.0, 7.1)	6.7 (6.1, 7.2)	6.3 (5.7, 7.0)	6.6 (5.9, 7.2)
*P-trend*	*0.045*	*0.027*	*0.997*	*0.914*	*<0.001*	*<0.001*	*0.104*	*0.488*

Note: SES, socioeconomic status. Gender-specific means (CI95) obtained by multivariable linear regression analysis adjusting for energy intake (continuous), baseline age (continuous), concurrent marital status (categorical) (model A), then for SES (education, social class and home-ownership) (model B). Model B numbers were: money for needs (8413); insufficient money for food/clothing (8417); difficulty paying bills (8425).

**Table 5:** Adjusted mean quantity and variety of vegetable intakes by financial hardship among older adults in the EPIC-Norfolk study.

	Vegetable quantity				Vegetable variety			
	Women		Men		Women		Men	
	Model A	Model B: + SES	Model A	Model B: + SES	Model A	Model B: + SES	Model A	Model B: + SES
**Enough money for needs**								
More than enough	279 (271, 287)	276 (268, 285)	256 (247, 265)	253 (244, 262)	17.0 (16.7, 17.2)	16.5 (16.2, 16.7)	16.6 (16.3, 16.9)	16.1 (15.9, 16.4)
Just enough	285 (281, 289)	286 (282, 290)	261 (256, 266)	262 (257, 267)	16.5 (16.4, 16.6)	16.6 (16.5, 16.7)	15.9(15.7, 16.0)	16.0 (15.8, 16.1)
Less than enough	280 (268, 292)	279 (266, 291)	262 (250, 275)	265 (252, 278)	15.9 (15.6, 16.3)	16.4 (16.0, 16.7)	15.2 (14.8, 15.6)	15.6 (15.2, 16.0)
*P-trend*	*0.904*	*0.776*	*0.396*	*0.106*	*<0.001*	*0.545*	*<0.001*	*0.057*
**Frequency of insufficient money for food/clothing**								
Never	281 (277, 286)	281 (276, 285)	260 (255, 264)	259 (254, 264)	16.6 (16.5, 16.8)	16.5 (16.3, 16.6)	16.0 (15.9, 16.2)	15.9 (15.8, 16.1)
Seldom	287 (279, 294)	289 (281, 297)	262 (254, 270)	263 (255, 272)	16.7 (16.4, 16.9)	16.8 (16.6, 17.1)	16.0 (15.7, 16.3)	16.1 (15.9, 16.4)
Sometimes	286 (276, 296)	288 (278, 298)	262 (251, 274)	264 (251, 276)	16.3 (16.0, 16.6)	16.7 (16.4, 17.0)	15.8 (15.4, 16.2)	16.2 (15.8, 16.5)
Often/Always	283 (267, 299)	284 (267, 301)	246 (226, 266)	252 (231, 273)	15.6 (15.1, 16.1)	16.0 (15.5, 16.5)	14.1 (13.5, 14.7)	14.6 (14.0, 15.3)
*P-trend*	*0.884*	*0.750*	*0.183*	*0.470*	*<0.001*	*0.054*	*<0.001*	*<0.001*
**Difficulty paying bills**								
None	282 (278, 287)	282 (278, 287)	258 (253, 263)	258 (253, 263)	16.5 (16.4, 16.7)	16.4 (16.3, 16.6)	16.0 (15.8, 16.1)	15.9 (15.7, 16.1)
Very little	287 (280, 294)	288 (280, 295)	265 (257, 273)	266 (258, 274)	16.8 (16.6, 17.1)	16.9 (16.7, 17.1)	16.0 (15.8, 16.3)	16.1 (15.9, 16.4)
Slight	290 (277, 303)	294 (280, 307)	259 (244, 274)	260 (245, 275)	16.5 (16.1, 16.9)	16.8 (16.4, 17.2)	16.2 (15.7, 16.6)	16.3 (15.8, 16.7)
Some	272 (259, 285)	273 (260, 286)	252 (237, 268)	255 (238, 271)	16.0 (15.6, 16.4)	16.4 (16.0, 16.9)	15.4 (14.9, 15.9)	15.8 (15.3, 16.3)
Great/Very great	283 (254, 312)	283 (253, 314)	278 (242, 313)	286 (249, 323)	15.4 (14.5, 16.4)	15.8 (14.9, 16.7)	14.0 (12.9, 15.1)	14.6 (13.5, 15.7)
*P-trend*	*0.635*	*0.652*	*0.453*	*0.213*	*0.001*	*0.054*	*<0.001*	*0.019*

Note: SES, socioeconomic status. Gender-specific means (CI95) obtained by multivariable linear regression analysis adjusting for energy intake (continuous), baseline age (continuous), concurrent marital status (categorical) (model A), then for SES (education, social class and home-ownership) (model B). Model B numbers were: money for needs (8413); insufficient money for food/clothing (8417); difficulty paying bills (8425).

Further adjustment for SES minimally attenuated inverse associations of FH with fruit variety (Table 4, model B), or vegetable variety (Table 5, model B). Differences by FH in mean fruit variety appeared stronger for women, after considering standard SES indicators. However, differences by FH in mean vegetable variety lost significance after additional adjustment, with two exceptions in men (frequency of not having enough money to afford adequate food/clothing and difficulty paying bills). Differences between the highest and lowest mean scores for combined FV variety were generally greater in men for each FH measure, overall and independent of SES (Appendix S1C).

Sensitivity analyses of other lifestyle factors showed similar results. Additional adjustment for quantity did not alter the relationship of any economic factor and variety of fruits or vegetables. Additional adjustment for variety in analyses of quantity either attenuated or amplified inverse associations with each economic exposure with no clear pattern for either gender. The same pattern of associations was also observed after excluding those reporting poor or moderate health (results available on request).

## Discussion

Quantity and variety of fruit and/or vegetable intakes were differentially associated with multiple economic conditions. Clear differences in variety of fruit and/or vegetable intakes were observed across three conventional SES indicators and three types of FH, whereas inverse associations for quantity outcomes were less consistent. For conventional SES indicators, differences in fruit variety and vegetable variety appeared larger in men. By contrast, differences by FH appeared somewhat larger in women for fruit variety and in men for vegetable variety. Among the different hardships, difficulty paying bills showed the greatest difference in mean variety between extreme categories. Relationships between FH and variety were independent of SES; conversely, differences in variety by SES were independent of FH.

The finding of differential social patterning between quantity and variety of fruit or vegetable consumption highlights the need to study them separately, given the separate health implications of variety and quantity for lowering disease risk (Cooper et al, 2012, Jeurnink et al, 2012). On the one hand, FV are low in energy and high in fibre and essential nutrients and minerals. Thus, *quantity* may benefit health by reducing the overall energy content of a diet, thereby improving its nutrient density (Cooper et al., 2012). On the other hand, *variety* of FV intakes will also have a specific role for health by ensuring a balance of the multitude of micronutrients, dietary fibre and other bioactive compounds necessary for maintaining physical functioning (Bernstein et al., 2002). Higher variety of FV intake might be particularly beneficial in terms of providing phytochemicals that are more specific to certain FV items that individuals with more varied intakes might consume preferentially (Randall, Nichaman, & Contant, 1985). For example, greater vegetable variety may provide individuals with specific sub-groups that contain high concentrations of flavonoids and carotenoids which have known health benefits (Mente, de Koning, Shannon, & Anand, 2009). Moreover, eating a wide variety of fruits or vegetables may also benefit health by ensuring a diverse composition of intestinal microbiota since diet-driven losses in the range of gut microbes are associated with increased frailty and health decline in older adults (Claesson et al., 2012).

While the biological mechanisms for the protective effects of variety are not fully elucidated, the health benefits of FV are likely to stem from both individual and synergistic effects of a range of nutritive and non-nutritive food components (Cooper et al, 2012, World Health Organization, 1998). Nutritional science would therefore benefit from research aimed at elucidating the unique health benefits of fruit variety and vegetable variety. Some suggest that a diet adequate in essential nutrients requires consuming a minimum of 15 different foods per week (Savige, Hsu-Hage, & Wahlqvist, 1997), but further work is also needed to establish what, if any, threshold of variety is needed *within* the fruit and vegetable food categories to support healthy ageing (McCrory et al., 1999). Despite a body of epidemiological evidence favouring a varied diet among older adults (Bernstein et al, 2002, Lee et al, 2011, World Health Organization, 1998), current recommendations remain limited in specifying thresholds for intakes across and within fruit and vegetable food groups (USDA & DHHS, 2010). They also lack clear distinction and emphasis on variety of intake which was more influenced by differences in economic conditions than quantity.

In the full EPIC-Norfolk cohort, social class and education level were independently associated with FV quantity, with educational differences stronger in women and social class differences stronger in men (Shohaimi et al., 2004). In the present sample of EPIC over-50s, significant associations of education and social class were found with fruit quantity for both genders, after considering financial hardship, that were consistent with wider literature (Pomerlau et al., 2008). For vegetable quantity, independent associations were borderline significant for education in men and wealth in women. Independent associations between SES and variety of either fruits or vegetables were significant in both genders, although SES differences were somewhat stronger in men which have no clear explanation. Moreover, compared to men, older women's fruit variety was more strongly associated with each of the novel FH measures which was consistent with known gender differences in the worse financial situation and stronger impact in women (Denton, Prus, & Walters, 2004).

Independent associations of FH with measures of healthful eating were also reported in a Finnish occupational study which examined overall FH in relation to a score of food habits recommended as healthy, and adjusted for similar SES indicators (Lallukka et al., 2006). Wider consumer economic literature also supports the observation that FH was independently associated with variety, more than quantity, of fruits and/or vegetables. Several consumer studies showed that individuals who shop for food under financial pressure tend to economise by limiting the variety of products before reducing the quantity of foods purchased, with the cheapest food items chosen within each food category (Wiig & Smith, 2009). We had expected everyday financial troubles to show stronger associations with FV intakes than SES because they would plausibly exert a more direct influence on decisions to purchase FV. Yet, in this cohort, conventional SES indicators showed slightly larger differences in mean variety of fruits and/or vegetables than those observed for FH measures. This finding might be explained by a phenomenological difference between SES and FH as the latter might have a relatively more transient nature than more time-invariant factors such as education, social class or home-ownership. Nonetheless, FH measures offered additional explanatory power for understanding variation in fruit variety and vegetable variety among older women and men as inverse associations remained significant after SES adjustment. Others have also reported independent effects of FH on weight gain (Loman, Lallukka, Laaksonen, Rahkonen, & Lahelma, 2013), which has known associations with FV consumption (Buijsse et al, 2009, Mozaffarian et al, 2011).

Given independent associations between SES, or FH, and healthful eating, there are likely two sets of pathways linking economic circumstances to diet quality among older people. Overall, SES may influence FV consumption through mechanisms involving dietary knowledge and health literacy, as well as social roles and cultural norms related to health and nutrition (Lumbers & Raats, 2006). In addition, through greater awareness of/exposure to the range of FV products, higher SES groups may have psychological attributes that may predispose them to favour new or unfamiliar types of foods in the FV categories (Eertmans, Baeyens, & Van den Bergh, 2001). Concurrently, FH may influence variety of FV intake through mechanisms that also involve material resources and spending power (Darmon & Drewnowski, 2008). Both vegetable variety and overall diet diversity are associated with higher diet cost (Keim et al, 2014, Lo et al, 2012). Thus, FH differences observed for variety of FV may be explained by the cost constraint of more expensive items (Rao, Afshin, Singh, & Mozaffarian, 2013). Older individuals with greater financial hardships, such as difficulty paying bills, might avoid more costly diverse diets because a higher proportion of their budgets is needed for housing and utility costs (Temple, 2006).

Preliminary analysis of dietary intakes reported at entry by the same over-50s indicated a difference of £0.62/day (16%) and £0.86/day (23%) in mean diet cost between the highest and lowest tertiles of fruit variety and vegetable variety, respectively (adjusted for age, gender and total energy intake). The role of price is potentially universal, as results from an RCT in New Zealand indicated no variation by ethnicity, income or education in the association of price discounts with purchasing of healthful foods (Blakely et al., 2011). Moreover, studies of older Australians and Taiwanese found greater food variety was associated with higher food expenditure, although associations were not adjusted for known confounders, particularly total energy intake (Lo et al, 2012, Temple, 2006).

Future research should formally explore potential mediators to determine shared and separate pathways that link SES, or hardship, with healthful eating using gender-specific analyses. Given that older adults are especially vulnerable to FH for multiple reasons (Kahn & Pearlin, 2006), efforts to promote FV consumption among older adults may benefit from a greater consideration of their everyday financial situation. Reducing financial barriers to FV variety is essential for older people whose greater need to manage chronic illnesses with healthful diets (Nolte & McKee, 2008) imposes a higher cost (Rao et al., 2013). Strategies might focus on helping their management of bill payment, and on improving reach to seniors of existing financial assistance programs (FRAC website).

Some study weaknesses are acknowledged. Exposure and outcome variables were self-reported and may be subject to recall or social desirability bias. Interpretation of the meaning of FH can also vary widely across a population; equivalent levels of financial strain can be perceived and experienced as status quo for some groups but as deprivation for others (Kahn & Pearlin, 2006). Thus, participants' responses about their economic conditions, either positive or negative, may be systematically influenced by an overall view of life. Nevertheless, precedent exists for the FH measures used here as findings of independent associations were consistent with studies of other health outcomes in similarly-aged groups (Kahn, Pearlin, 2006, Laaksonen et al, 2011). Furthermore, subjective levels of FH deserve investigation as perceived resources might impact diet variety more than actual levels in older age (Dean, Raats, Grunert, & Lumbers, 2009). Hardship was measured once approximately 18 months before diet; thus durations or transitions could not be ascertained. There may therefore be misclassification of exposures stemming from changes to participants' hardship levels in the interval between surveys. However, this would have biased results towards the null since any misclassification would be unrelated to dietary outcomes and thus non-differential. It is possible that low FV intake contributed to poor health which might lead to low SES or high FH. Our exclusion of over-50s reporting poor or moderate health at cohort entry did not suggest that independent associations were changed.

Results may be subject to residual confounding from income, which was not collected in the cohort, since low income has been associated with low FV consumption in older adults (Bihan et al., 2010). While the unobserved influence of income cannot be discounted, current income is not consistently associated with diet quality and does not fully characterise a person's financial situation; nor is it the only structural resource used by older adults to fund their expenses (Irz et al., 2013). Residual confounding is also possible from not examining other types or functions of social relationships (e.g. existence of a trusted confidant) that can be important factors influencing diet quality (Locher et al., 2005), or FV variety (Conklin et al., 2013), and might also contribute to SES-based health inequalities (Stringhini et al., 2012). Future research should explore how both social and economic aspects of older individuals' life circumstances interact to influence dietary behaviours as called for in the public health research and policy literature (Killoran & Kelly, 2010).

Notwithstanding some limitations, this study has several strengths: a large sample size, gender-specific analyses, adjustment for known confounders including multiple lifestyle factors, and six factors describing older people's economic conditions. Multiple economic factors were examined, including potentially important variables of the financial situation. The examination of three separate FH measures was important for providing unique information on whether different types of this economic domain might be associated with diet quality (Turrell, Hewitt, Patterson, & Oldenburg, 2003). This study also included a proxy for wealth which was employed as a unique SES measure since a review of evidence on SES indicators showed home-ownership was a useful measure of wealth in older populations (Pollack et al., 2007), and wealth is known to be associated with diet in UK elderly (Maynard et al., 2006). Additionally, it further specified as many relevant economic factors as possible (rather than SES overall) for women and men separately to avoid assuming economic comparability of individuals who are similar on, for example, education (Braveman et al., 2005). As recommended, it used multiple categories for specified economic factors which helped to uncover important differences in diet quality that could apply across the social spectrum (Braveman et al., 2005). Finally, we examined multiple economic influences on two separate measures of healthful eating. Variety of foods, specifically FV intake, has several unique attributes: it is a good marker of overall diet quality (Bernstein et al, 2002, Drewnowski et al, 1997, Keim et al, 2014); counting the number of different fruit and vegetable items has shown utility for chronic disease aetiology (Cooper et al, 2012, Jeurnink et al, 2012), and, it is an established concept in dietary recommendations (USDA & DHHS, 2010), including for older adults (World Health Organization, 1998).

### Conclusion

To conclude, this study found that variety, more than quantity, of FV intake among older adults in this UK cohort was consistently patterned by diverse economic conditions. Different types of FH each provided additional explanatory power for understanding variation in the variety of FV consumed beyond education, social class and home-ownership. However, differences by SES appeared somewhat stronger. Health promotion and interventions to increase FV consumption among older adults will need to explicitly call out the importance of variety and focus on improving fruit variety in women and vegetable variety in men. Strategies are needed to address the financial barriers that might limit the uptake of such dietary advice.

## Appendix Supplementary material

Appendix S1 Appendix S1A–C

## Footnotes

Appendix Supplementary data to this article can be found online at doi:10.1016/j.appet.2014.08.038.
