# Supplementary material for: Variety more than quantity of fruit and vegetable intake varies by socioeconomic status and financial hardship. Findings from older adults in the EPIC cohort
Source: Appetite. 2014 Dec 1;83:248–55. doi: 10.1016/j.appet.2014.08.038 (PMC4217146; doi:10.1016/j.appet.2014.08.038)
Supplement: Appendix S1 — A–C [file mmc1.docx]

**Variety more than quantity of fruits and vegetable intakes varies by socioeconomic status and financial hardship. Findings from older adults in the EPIC cohort**

**Appendix A** The process of sample selection from the EPIC-Norfolk cohort

**
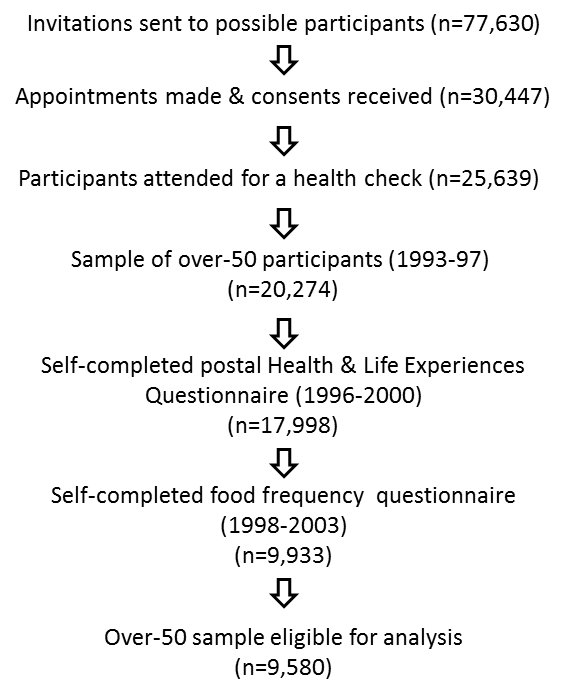
**

**Appendix B** Adjusted mean quantity and variety of combined fruits and vegetables by socioeconomic status among older adults in the EPIC-Norfolk study

|  |  | **Fruit and vegetable quantity^b^** | |  | **Fruit and vegetable variety^b^** | |
| --- | --- | --- | --- | --- | --- | --- |
|  |  | **Women** | **Men** |  | **Women** | **Men** |
| *Social Class^c^* | | | | | | |
| Professional |  | 595  (569, 621) | 524  (497, 550) |  | 25.6  (25.1, 26.2) | 24.6  (24.0, 25.1) |
| Managerial and Technical |  | 596  (585, 607) | 509  (498, 521) |  | 26.3  (25.0, 25.5) | 23.7  (23.4, 23.9) |
| Skilled non-manual |  | 572  (557, 587) | 488  (468, 508) |  | 23.9  (23.5, 24.2) | 22.2  (21.8, 22.7) |
| Skilled manual |  | 581  (566, 596) | 480  (464, 496) |  | 23.4  (23.1, 23.7) | 21.4  (21.0, 21.7) |
| Partly skilled |  | 566  (546, 585) | 484  (463, 505) |  | 23.3  (22.9, 23.7) | 21.1  (20.6, 21.5) |
| Unskilled |  | 563  (527, 599) | 455  (406, 505) |  | 22.1  (21.4, 22.9) | 20.4  (19.3, 21.4) |
| *Education* | | | | | | |
| Degree |  | 598  (578, 618) | 524  (505, 543) |  | 26.0  (25.5, 26.4) | 24.6  (24.2, 25.0) |
| A-level |  | 593  (582, 604) | 496  (485, 507) |  | 25.1  (24.9, 25.3) | 22.8  (22.6, 23.1) |
| O-level |  | 567  (547, 587) | 492  (467, 517) |  | 24.4  (24.0, 24.9) | 23.0  (22.5, 23.6) |
| No qualification |  | 573  (563, 584) | 483  (469, 497) |  | 23.0  (22.7, 23.2) | 21.1  (20.8, 21.4) |
| *Home-ownership* | | | | | | |
| Owner-occupier |  | 583  (576, 590) | 499  (490, 507) |  | 24.4  (24.3, 24.6) | 22.8  (22.7, 23.0) |
| Renting, private |  | 576  (532, 620) | 470  (421, 520) |  | 23.2  (22.2, 24.1) | 21.3  (20.3, 22.4) |
| Renting, public |  | 565  (536, 594) | 475  (434, 513) |  | 21.8  (21.2, 22.4) | 19.8  (19.0, 20.6) |

Gender-specific means (CI95) obtained by multivariable linear regression analysis adjusted for energy intake (kcal/d), baseline age (continuous), and concurrent marital status (married/cohabiting, single, widowed, divorced/separated). Numbers analysed were: social (n=9,365); education (9,531); home-ownership (n=8,661). ^b^ Quantity was a continuous variable summing the total amount of each product reported as consumed (g/d); variety was a continuous score summing the total number of different products consumed in any quantity over a month or less (items/m). ^c^ Based on UK Registrar General’s hierarchy of occupations to derive social classes.

**Appendix C** Adjusted mean quantity and variety of combined fruits and vegetables by financial hardship among older adults the EPIC-Norfolk study

|  | **FRUIT AND VEGETABLE QUANTITY^b^** | | | |  | **FRUIT AND VEGETABLE VARIETY^b^** | | | |
| --- | --- | --- | --- | --- | --- | --- | --- | --- | --- |
|  | **Women** | | **Men** | |  | **Women** | | **Men** | |
|  | ***Model A*** | ***Model B: +SES*** | ***Model A*** | ***Model B: +SES*** |  | ***Model A*** | ***Model B: +SES*** | ***Model A*** | ***Model B: +SES*** |
| *Enough money for needs* | | | | | | | | | |
| More than enough | 577  (561, 593) | 570  (554, 587) | 497  (479, 514) | 489  (471, 507) |  | 24.9  (24.6, 25.3) | 24.3  (23.9, 24.6) | 23.7  (23.3, 24.1) | 23.1  (22.7, 23.4) |
| Just enough | 583  (575, 591) | 586  (577, 594) | 496  (487, 505) | 500  (490, 509) |  | 24.2  (24.0, 24.4) | 24.4  (24.2, 24.5) | 22.5  (22.3, 22.7) | 22.7  (22.5, 22.9) |
| Less than enough | 576  (552, 599) | 574  (549, 599) | 498  (473, 524) | 502  (476, 528) |  | 23.2  (22.6, 23.7) | 23.7  (23.2, 24.2) | 21.6  (21.0, 22.1) | 22.1  (21.6, 22.7) |
| *Frequency of not having enough money for food or clothing* | | | | | | | | | |
| Never | 582  (573, 591) | 581  (572, 590) | 500  (490, 510) | 499  (489, 509) |  | 24.4  (24.2, 24.6) | 24.2  (24.0, 24.4) | 22.8  (22.6, 23.1) | 22.7  (22.5, 22.9) |
| Seldom | 584  (569, 599) | 588  (573, 603) | 494  (477, 510) | 499  (482, 516) |  | 24.5  (24.2, 24.8) | 24.7  (24.4, 25.0) | 22.8  (22.4, 23.1) | 23.0  (22.7, 23.4) |
| Sometimes | 585  (565, 604) | 586  (566, 606) | 488  (464, 511) | 491  (467, 515) |  | 23.8  (23.4, 24.3) | 24.3  (23.9, 24.7) | 22.4  (21.9, 22.9) | 22.9  (22.4, 23.4) |
| Often/ Always | 550  (518, 583) | 549  (515, 583) | 478  (438, 518) | 481  (440, 523) |  | 22.5  (21.8, 23.2) | 23.1  (22.4, 23.8) | 20.2  (19.3, 21.1) | 20.9  (20.0, 21.8) |
| *Difficulty paying bills* | | | | | | | | | |
| None | 585  (576, 594) | 584  (575, 593) | 499  (489, 509) | 500  (490, 510) |  | 24.3  (24.1, 24.5) | 24.2  (24.0, 24.4) | 22.7  (22.5, 22.9) | 22.6  (22.4, 22.9) |
| Very little | 580  (565, 595) | 581  (566, 596) | 497  (481, 513) | 500  (484, 516) |  | 24.7  (24.4, 25.0) | 24.8  (24.5, 25.1) | 22.8  (22.5, 23.1) | 23.0  (22.6, 23.3) |
| Slight | 589  (563, 615) | 594  (568, 621) | 485  (455, 514) | 485  (455, 515) |  | 24.0  (23.5, 24.6) | 24.4  (23.9, 25.0) | 23.1  (22.4, 23.7) | 23.2  (22.6, 23.8) |
| Some | 560  (535, 586) | 564  (537, 590) | 469  (438, 500) | 474  (442, 507) |  | 23.4  (22.8, 23.9) | 24.0  (23.4, 24.5) | 21.8  (21.1, 22.5) | 22.4  (21.7, 23.0) |
| Great/ Very great | 543  (485, 601) | 535  (475, 595) | 526  (455, 597) | 530  (456, 603) |  | 22.0  (20.8, 23.3) | 22.5  (21.2, 23.7) | 20.3  (18.8, 21.9) | 21.2  (19.6, 22.7) |

Gender-specific mean (CI95) quantity (g/d) or variety (items/m) of fruits and vegetables obtained by multivariable linear regression models adjusting for energy intake (continuous), baseline age (continuous), concurrent marital status (categorical) (Model A), then socioeconomic status (SES) (Model B). Model B numbers were: money for needs (n=8,413); not having enough money (n=8,417); difficulty paying bills (n=8,425). ^b^ Quantity was a continuous variable summing the total amount of each product reported as consumed (g/d); variety was a continuous score summing the total number of different products consumed in any quantity over a month or less (items/m).
